# Failure

**Published:** 1890-09-06

**Authors:** 


### Words of Consolation.

### FAILURE.

We all think in moments of depression that we are complete failures, we have done nothing in our lives worthy of notice, and now that we are sick or afflicted we can never do anything for the benefit of mankind. We quite enter into the saying of the Wise Man, who, in Ecclesiasticus declares, "Some there be which have no memorial; who are perished as though they had never been; and are become as though they had never been born; and their children after them." The same lesson is taught by the same writer comparing life to an arrow which passes swiftly through the air and leaves no trace of its path, or of a vessel round whose keel the waters close and the track is immediately lost.

But why should we complain when very little is known of the greatest men, or even of the Apostles, except SS. Peter, Paul, and John, and those few whose writings reveal their inner life? Of the rest their characters, works, and labours for Christ are lost to fame. God alone saw and remembers their battles with sin, the world, and the temptations of the flesh. "Their lives are hid with God in Christ." And it should be much consolation to us to know that it is so. It is not the great, the noble, the wealthy who are praised and flattered, and talked about, who are the happiest in this life or the highest in the Kingdom of Heaven. Here they have their trials and their vexations like other folk, and when they are dead "six feet of earth ends all distinction of their birth."

It is a useful lesson, then, at all times to remember the fact that so many true Saints of God have left no memorial. If we ourselves have hardly been heard of by our neighbours, let us thank God if only we may be found to be obscure disciples of Jesus. Our good deeds may be but a faint light shining in a naughty world; and so long as we remember Christ's command not to let our left hand know what our right hand doeth, we know that our best actions should only redound to God's glory, not to our own. How quietly the Great Creator formed the world! "In silence and in shade, earth's foundations first were laid." His Spirit brooded over the waters and with a word life and light sprang into being. Christ's dealings with man's soul are similar. We hardly know when His Holy Spirit begins to work in our hearts, but suddenly the full glow of His love bursts out, and we feel that we are His for time and for Eternity. Of that "Love which boasts a heavenly birth," the poet says—

So still and secret in her growth,
Ever the truest heart,
Where deepest strikes her kindly root
For hope or joy, for flower or fruit,
Least knows its happy part.

